# Digit ratio in the common toad *Bufo bufo*: the effects of reduced fingers and of age dependency

**DOI:** 10.1186/s40851-021-00174-y

**Published:** 2021-03-25

**Authors:** Mikołaj Kaczmarski, Jan M. Kaczmarek, Łukasz Jankowiak, Krzysztof Kolenda, Piotr Tryjanowski

**Affiliations:** 1 Institute of Zoology, Poznań University of Life Sciences, Wojska Polskiego 71c, PL 60-625 Poznań, Poland; 2 Institute of Biology, University of Szczecin, Wąska 13, PL 71-415 Szczecin, Poland; 3 Amphibian Biology Group, Department of Evolutionary Biology and Conservation of Vertebrates, Institute of Environmental Biology, University of Wrocław, Sienkiewicza 21, PL 50-335 Wrocław, Poland; 4 Faculty of Environmental Sciences, Czech University of Life Sciences Prague, Kamýcká 129, 165 00 Prague 6, Czech Republic

**Keywords:** Anura, Morphology, Sexual dimorphism, 2D:3D, 2D:4D, 3D:4D, Digit identity

## Abstract

**Introduction:**

Despite the growing number of studies describing digit ratio patterns in tetrapods, knowledge concerning certain basic issues is still scarce. In lower vertebrates such as tailless amphibians (Anura), the numbering of individual fingers on the forelimbs and their homology with the fingers of other vertebrates pose an unsolved problem. Based on reviewed data on anuran limb development, we argue that the correct finger numbering scheme should be based on the assumption that the first finger, not the fifth finger, was reduced on the forelimbs. We analyzed the digit ratio in the common toad (*Bufo bufo*, Bufonidae), a species characterized by well-developed sexual dimorphism whereby females are larger than males, using both numbering schemes present in the literature.

**Results:**

We found that the digit ratio on hindlimbs differed significantly between the sexes only in the cases of left 2D:3D, with lower digit ratios in females, and of left 3D:4D, with lower digit ratios in males. We found that sex was the only significant variable for forelimbs, differentiating 2D:3D on the left forelimb, with lower digit ratios in females; 2D:4D on the right forelimb, with lower digit ratios in males; and 3D:4D on both forelimbs, with lower digit ratios in males. These results relate to variant II reflecting the hypothesis that the first digit was reduced during phylogenesis. There was no relationship between the body size (SVL) of individuals and any digit ratio, excluding 2D:4D on the right forelimbs in models with age variables. Additionally, for a subset of data where individual age was known, the models indicated that age was linked to significant differences in 2D:4D and 3D:4D on the left hindlimbs, while age, SVL, and sex influenced 2D:4D on the right forelimbs.

**Conclusion:**

We emphasize the importance of the problem of the correct numbering of forelimb digits in Anura and, under the assumption that it was the fifth digit that was reduced, argue that earlier results on digit ratio in this group should be interpreted with caution. The detected relationship between digit ratio and age in amphibians expands our knowledge, indicating that the age of individuals should be included in future digit ratio studies. This relationship may also apply to studies using digit ratio as a noninvasive indicator of endocrine disruption in amphibians.

**Supplementary Information:**

The online version contains supplementary material available at 10.1186/s40851-021-00174-y.

## Background

Digit ratio (2D:4D) denotes the relative lengths of the second and fourth digits. This ratio appears to be correlated with levels of sex hormones during development [1, 2]. The number of studies examining the relationships between digit ratio and behavioral or physical features is growing dynamically, especially in the case of *Homo sapiens* [3, 4]. Most of these are correlation studies, but in the case of vertebrates other than humans, experimental work has also been carried out, shedding new light on the evolution of the digit ratio pattern in tetrapods [5–12]. It is generally accepted that 2D:4D is higher in females; this is true not only for most mammals [13–16] but also for most tailed amphibians (newts [17];). The opposite pattern is present in most sauropsids (i.e., birds and reptiles) [18–21]. However, at the same time, a growing number of studies have been unable to confirm these patterns in various tetrapod lineages or have detected patterns different than expected [22–24].

### Digit ratio in amphibians: current state of knowledge

Amphibians have been studied the least among modern tetrapods in terms of digit ratio patterns. Most existing studies are descriptive, with the exception of one experimental study in which testosterone levels were manipulated during development [5]. We are currently aware of seven species within Anura [5, 24–27] and five within Caudata [17, 28] in which digit ratio studies have been conducted (for more details, see Table 1). A male-biased pattern, i.e., males characterized by a significantly higher 2D:4D than females, was described for the hindlimbs in the pointed-belly frog *Leptodactylus podicipinus* and in the strawberry poison-dart frog *Oophaga pumilio* [5, 25]. However, a subsequent study of *O. pumilio* did not confirm the earlier results: among the examined individuals, males were characterized by a significantly higher 2D:4D on the forelimbs than females, while no differences between the sexes were detected for the hindlimbs [26]. Similarly, in Bransford’s robber frog *Craugastor bransfordii*, males were characterized by a higher 2D:4D than females, but this was true only for the left forelimbs [26]. On the other hand, in the túngara frog *Engystomops pustulosus*, females were characterized by a higher 2D:4D than males for both forelimbs, a pattern considered characteristic of mammals [27]. A similar phenomenon was observed in the marsh frog *Pelophylax ridibundus* for the left forelimb; however, only the left side of the body was examined in this study [29]. Female-biased patterns have also been detected for the hindlimbs in Salamandridae, specifically in the smooth newt *Lissotriton vulgaris*, the Carpathian newt *Lissotriton montandoni*, and the alpine newt *Ichthyosaura alpestris* [17]. In the last species, this pattern was additionally recorded for the forelimbs [17]. However, in two other Caudata species, the great crested newt *Triturus cristatus* and the fire salamander *Salamandra salamandra*, no sex differences were found in the digit ratios of any limbs [17, 28]. This is also true for the Maud Island frog *Leiopelma pakeka*, the cane toad *Rhinella marina*, and the rufous frog *Leptodactylus fuscus* [5, 24, 27] (Table 1).

**Table 1:** A review of available publications on the most frequently investigated digit ratio (2D:4D) in amphibians, with the main results and additional information. Results for which significant sexual dimorphism in digit ratio was detected are indicated in bold. The effect sizes (to two decimal places) are included in parentheses where reported in parentheses. For values of other digit ratios (2D:3D and 3D:4D) in the common toad *Bufo bufo*, see Tables 2, 3, and S2

Order	Sample size		Adult SVL	Specimen type	2D:4D				References
Family, *Species*	M	F			FL	FR	BL	BR	
**Anura**									
Bufonidae									
*Bufo bufo*^a^	158	143	F > M	Live	ns	ns			this study
					(−0.08)	(0.02)	ns	ns	
					ns^#^	**M < F**^**#**^	(− 0.08)	(− 0.01)	this study
					(0.15)	**(−0.53)**			
*Rhinella marina*	24	28	F > M	Live	ns	ns	ns	ns	[27]
*R. marina*	14	5	F > M	Live	ns	ns	ns	ns	[27]
Craugastoridae									
*Craugastor bransfordii*	46	42	F > M	Live	**F < M**	ns	ns	ns	[26]
					**(0.62)**	(0.12)	(0.33)	(0.1)	
Dendrobatidae									
*Oophaga pumilio*	40	44	F = M	Live	ns	ns	**F < M**	**F < M**	[25]
*O. pumilio*	36	48	F = M	Live	**F < M**	**F < M**	ns	ns	[26]
					**(0.93)**	**(0.7)**	(0.05)	(0.2)	
Leiopelmatidae									
*Leiopelma pakeka*			F > M	Live	–	ns	–	ns	[24]
Leptodactylidae									
*Engystomops pustulosus*	107	52	F > M		**M < F**	**M < F**	ns	ns	[27]
*Leptodactylus podicipinus*^b^	128	191	F > M	Preserved	–	–	**F < M**	**F < M**	[5]
*Leptodactylus fuscus*^c^	24	25	F = M	Live	–	–	ns	ns	[5]
Ranidae									
*Pelophylax ridibundus*^c^	30	20	F > M	Live	**M < F**	–	ns	–	[29]
**Caudata**									
Salamandridae									
*Salamandra salamandra*	35	31	F > M	Preserved	ns	ns	ns	ns	[28]
*Triturus cristatus*	25	20	F = M	Preserved	ns	ns	ns	ns	[17]
					(−0.18)	(−0.3)	(−0.2)	(−0.18)	
*Ichthyosaura* (*Mesotriton*) *alpestris*	42	39	F > M	Preserved	**M < F**	**M < F**	**M < F**	**M < F**	[17]
					**(−0.34)**	**(−0.37)**	**(− 0.48)**	**(− 0.33)**	
*Lissotriton montandoni*	42	33	F > M	Preserved	ns	ns	**M < F**	**M < F**	[17]
					(−0.01)	(− 0.03)	**(− 0.26)**	**(−0.32)**	
*Lissotriton vulgaris*	42	41	F > M	Preserved	ns	ns	**M < F**	**M < F**	[17]
					(−0.13)	(−0.03)	**(− 0.4)**	**(− 0.29)**	

Abbreviations: *M* Male, *F* Female; *SVL* Snout-vent length; *FL* Left forelimbs, *FR* Right forelimbs, *BL* Left hindlimbs, *BR* Right hindlimbs, *ns* No significant differences. Number of study populations: ^a^ – three populations, ^b^–6 populations; ^c^ – two populations. # - variant II, according to numbering protocol for forelimb fingers reflecting the hypothesis that the first digit was reduced during phylogenesis in Anura. In other cases, the digit ratio was calculated for variant I (reflecting that the fifth digit was reduced) [24–27, 29]. For more details see Fig. 1

A recent experimental study on *L. fuscus* made a significant contribution to the current understanding of the digit ratio (2D:4D) pattern in amphibians and, more broadly, tetrapods [5]. In the study, Gosner stages 28 to 46 (the end of metamorphosis) were considered a developmental window in which gonadal and digit development occurs in anurans. Consequently, it was assumed that developing tadpoles are susceptible to hormonal manipulation, which, hypothetically, should change the digit ratio pattern. The experiment involved the addition of testosterone to water in tanks with developing tadpoles. In line with the assumptions, digit ratios were sensitive to levels of sex steroids during ontogeny: individuals undergoing treatments with added testosterone exhibited a masculinized 2D:4D on the hindlimbs (more precisely, digit II (2D) became longer in frogs treated with testosterone, whereas no effect was detected for digit IV (4D)). The male-biased 2D:4D observed in *L. fuscus* was apparently a consequence of the varying level of sensitivity of 2D to circulating testosterone, which differed between the sexes—the “*hypothesis of changes in the identity of dimorphic digits*” [5]. The authors also pointed out that phenotypic integration occurs between traits such as digit ratio and body size, both of which are hormonally modulated by testosterone levels during larval development [5]. The conclusions drawn by these authors appear to be in line with those from previous results (see Table 1) and thus shed new light on the inconclusive results obtained heretofore in amphibians. Thus, the digit ratio pattern in a given case may depend to a large extent on the evolutionary history of the species and may be modified as a result of natural selection or the adaptation that species to its lifestyle and environmental conditions (see also the results obtained in connection with phylogenetic background by [30] for iguanian lizards).

Another study provided a more detailed understanding of the effect of the scaling relationships between the second and fourth digits on Anura limbs, using sex, body size, and body side as interactive fixed effects for data obtained from *E. pustulosus* and *R. marina* [27]. There were no significant differences between sexes in digit ratio, but it was found that in *E. pustulosus,* the length of 2D was best predicted by the interaction between body size and the length of 4D. In contrast, in *R. marinus,* there was no relationship between 2D:4D and sex or body size. However, larger toads are characterized by a longer 2D, the length of which was best predicted by the length of 4D, which was interpreted as isometric growth [27]. In conclusion, a body size component should be included in all digit ratio studies.

However, in the case of anuran amphibians, comparison of results concerning digit ratio patterns with those for other tetrapods leads to additional difficulties that have not been properly discussed. [27] were the first to indicate that, on forelimbs, “[the] digit numbering scheme used in this and other studies of anuran digit ratios may not be homologous to digit numbers used in other pentadactyl taxa.” This is because we are still not sure “which digit on the anuran forelimb was lost over evolutionary time from their pentadactyl ancestors” [27]. In earlier papers, digit numbering was adopted according to the first paper on digit ratio in amphibians, in which the author proposed that “[the] digit order was considered medial to lateral, in which the most medial was the ‘first’ digit” [25]. Later, other authors analyzed digit ratio on anuran forelimbs using the same schema [24, 26, 27, 29]. However, the numbering adopted by [25] conflicts with current knowledge concerning the biology of limb/toe development in anuran amphibians [31, 32]. According to experimental studies, the lost finger is the most preaxial finger, i.e., finger I [33]. As a consequence, anuran forelimbs most likely contain fingers II–V [31, 32].

**Fig. 1:**
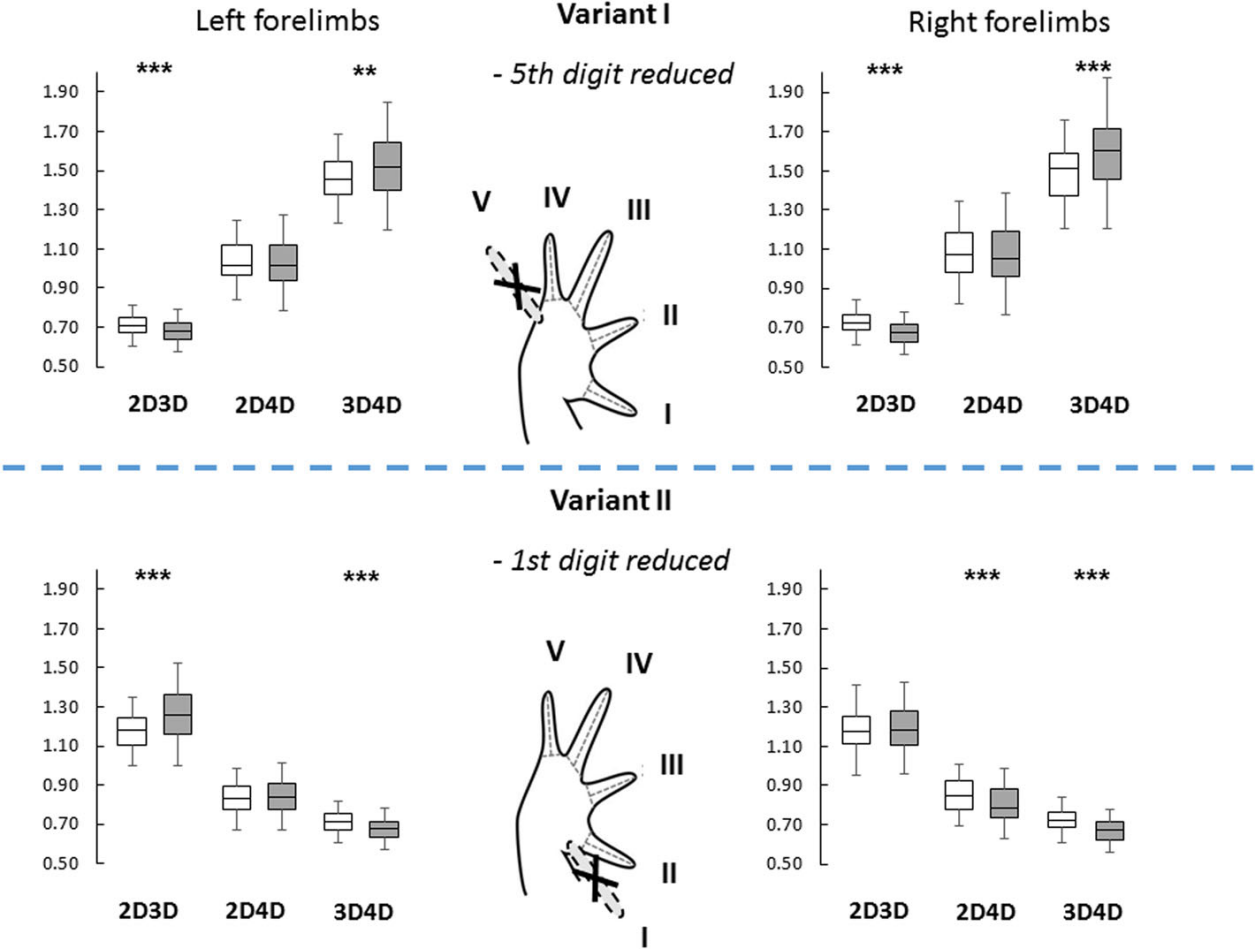
Two alternative finger numbering protocols for anuran forelimbs, reflecting the hypotheses that the fifth (variant I) or the first (variant II) digit was reduced during phylogenesis. The upper and lower parts of the figure show which fingers are used to measure digit ratios in each of the adopted protocols, with the results presented in the form of box plots. It should be noted that 2D:3D in variant I corresponds to 3D:4D in variant II; similarly, 2D:4D corresponds to 3D:5D, and 3D:4D corresponds to 4D:5D. Although variant I is predominantly used in research, it is variant II that probably reflects the actual finger numbering in anurans (see in text: Limb and finger development in anurans). Asterisks indicate the statistical significance level of the difference in digit ratios between sexes (gray box plots - male; white box plots – female; levels of significance: ***p < 0.001, **p < 0.01). The illustration of an anuran forelimb was adapted from [27]

The aim of our study was to investigate digit ratio (2D:4D and other described combinations, such as 2D:3D and 3D:4D) in the common toad *Bufo bufo* (Linnaeus, 1758) (Bufonidae), a species with pronounced sexual dimorphism, whereby females are much larger than males. We hypothesized that sex differences in digit ratio are present in this species but made no specific predictions about the direction of the pattern (female- or male-biased). Our results were analyzed using two alternative variants of digit numbering on the forelimbs (see Fig. 1 for more details). In variant I, following earlier researchers of the subject, we assumed that the fifth digit was lost during development ([25] and further studies). In variant II, we assumed that the first digit, i.e., the most preaxial one, was lost during development [33]. However, as we pointed out above, the digit numbering in variant I is not supported by current knowledge concerning forelimb growth patterns in amphibians [31, 32]. Additionally, we took individual age into account when analyzing the digit ratios of animals originating from one of the studied populations to determine whether potentially different environmental conditions during development lead to differences in digit ratios between age cohorts. Toads in the studied populations reach an age of approximately 4 years, with the few oldest individuals reaching ages over 5 years [34, 35], and we supposed that the age cohorts might differ in sexual dimorphism in digit ratios. Our assumption was based on the fact that environmental conditions (including environmental stressors) during larval development (i.e., the tadpole stage) may vary between years, leading to slightly different phenotypes of adult individuals between age cohorts. As a consequence, this variation may be reflected in detectable differences in digit ratios. To the best of our knowledge, in the existing digit ratio studies on adult amphibians, the age of the studied individuals was never included.

## Methods

### Ethics statement

This research complies with the current laws of Poland and was performed with appropriate collection and research permits (from the Regional Director of Environmental Protection: WPN.6401.57.2014.IW and WPN.6401.42.2014.MD.I.). We followed all applicable institutional and national guidelines for the care and use of animals. Moreover, the main investigator (MK) has been trained by the Polish Laboratory Animal Science Association. The study species, the common toad *Bufo bufo*, has been assigned ‘least concern’ status at the European Union level [36]; however, at the national level, the current status of this species has not been evaluated [37]. Once measurements had been taken, all animals were immediately released at the site of capture in accordance with the permission granted.

### Study sites and sampling

The study was carried out at three separate sites in western Poland: at two sites located within the city of Poznań (Krzesiny – site 1: 52.3370° N, 16.9795° E, Kajka – site 2: 52.2518° N, 16.5910° E) and one in a low-disturbance, forested landscape (Gorzyń – site 3: 52.5493° N, 15.8736° E). The first two sites were described in our earlier paper; both are subject to human impact, with anthropogenically altered habitats (urbanization, pollution, and road mortality were noted) [34]. In contrast, the population from site 3, which is relatively uninfluenced by human activity, inhabits a mixed forest. All individuals from each site were collected during a single night with pitfall traps or captured manually during a massive spring migration (March–April 2015). We analyzed a total of 299 *B. bufo* individuals (141 females, 158 males): 54 males and 50 females from site 1; 49 and 53 from site 2; and 55 and 40 from site 3. To avoid resampling of individuals, each location was sampled only once. The sex of individuals (all of the measured specimens were sexually mature) was determined using the occurrence of nuptial pads in males as well as differences in body size and shape.

Two characteristics were measured using a manual caliper (accuracy: 0.01 mm): SVL (snout-vent length, which in anurans is identical to total body length) and head width (HW). To minimize additional human-induced systematic error (observer effect), all specimens were measured by only one researcher. To calculate intraobserver error, digit measurements were carried out twice for 30 randomly selected individuals. The level of technical measurement error was calculated using the intraclass correlation coefficient (ICC).

### Digit ratio measurements

A special measurement platform was assembled so that all photographs could be taken from the same distance and would depict the same position of the digits. The platform consisted of a wooden frame with two glass surfaces placed 6 cm from each other. In each case, the limb and camera were installed in the frame; the limb was placed on the upper surface (lined with millimeter paper, which served as a scale), and the camera, on the lower surface (the opposite side). Separate pictures were taken for each fore- and hindlimb. Then, we used computerized measurements of each limb photograph, in accordance with [17]. Limbs with distorted and missing digits were excluded from our study.

### Age evaluation

In amphibians, growth parameters such as body length and weight are closely related to environmental factors and thus should not be used to estimate age [38]. Therefore, precise determination of the age of studied individuals requires the use of other methods. We evaluated the age of individuals from site 2 using skeletochronological analysis in accordance with the schema described by [39], with some modifications of the protocol (see [34] for details). Following digitalization of each limb, we cut off the fourth toe of the hindlimb of each individual using microsurgical scissors that were sterilized before each use. We disinfected the resulting wound on live toads with 0.01% potassium permanganate. Subsequently, toes were decalcified, sliced with a freezing microtome, and stained with cresyl violet. For age evaluation, we counted the number of LAGs deposited in periosteal bone during each hibernation (1 LAG = 1 hibernation = 1 year of life) using a Carl Zeiss Axioscope 20 light microscope (for more details, see our previous papers: [34, 35]).

### Data processing and statistical analysis

In our analyses, we used general linear models (GLMs). We tested the size (SVL) and sex of toads as the factors affecting digit ratio. In the first models, we used data from each site and built mixed models with the site of capture as a random variable (random intercept); however, in all models, the value of this parameter was zero, indicating no subject-level variation. We performed modeling on different response variables: left and right forelimb 2D:4D: variant I (n_left_ = 290, n_right_ = 290) and variant II (n_left_ = 287, n_right_ = 286) (for more details about variants, see Fig. 1.). An SVL value difference was observed between males and females (mean for males: 65.66 ± 5.08 sd; mean for females: 85.22 ± 7.16; t-value: − 27.26, df = 293, *p* <  0.001). To eliminate this difference, prior to these analyses, the SVL value for each individual was centered around the mean size for the specimen’s sex. In the second set (dataset 2) of models, we additionally tested the age variable for individuals from site 2 (for all 99 records). We compared each digit ratio linked to the most frequently investigated 2D:3D, 2D:4D, and 3D:4D (see Fig. 1) in both variants for the forelimbs and hindlimbs between the right and left sides, using a t-test for dependent variables. We used Hedges’ *g* statistic [40] to estimate the effect size. The magnitude of the effects was categorized using four thresholds: |*g*| <  0.2, “negligible”; 0.2 ≤ |*g*| <  0.5, “small”; 0.5 ≤ |*g*| <  0.8, “medium”; and 0.8 ≤ |*g*| < 1 “large” [41]. All of the statistics were calculated in R software [42] with the ‘lme4’ package [43]. The explanatory variables were tested using the drop1() function. We diagnosed models graphically but found no violation of either homoscedasticity or normality of residuals.

In accordance with the approach adopted by [30], we calculated the sexual dimorphism index for body size (SDI_SVL_) and selected digit ratios (SDI_digit ratio_), i.e., those significantly influenced by age and sex. The indexes were evaluated in the context of phenotypic integration as described by [5].

## Results

**Fig. 2:**
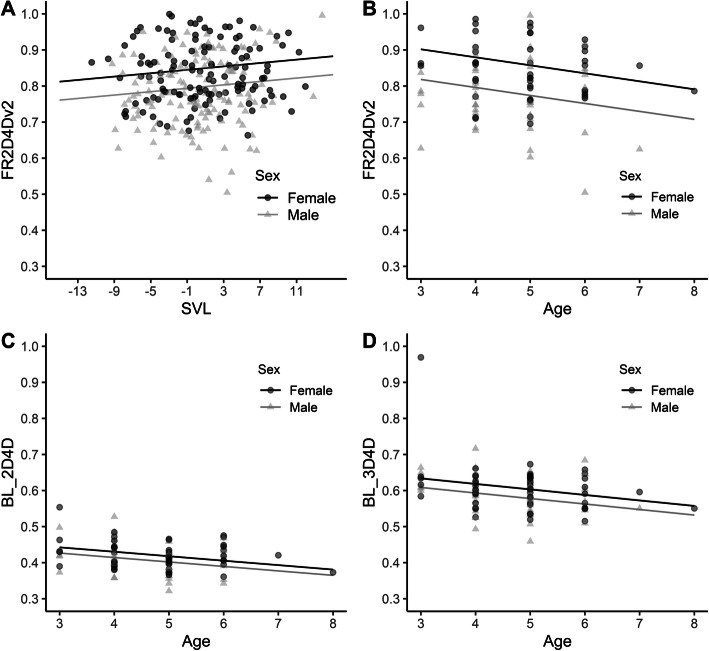
Panel **a**: relationship between size (SVL, snout-vent length) of the common toad *Bufo bufo* and right forelimb 2D:4D (variant II) for each sex (for each individual, SVL was centered around the mean size for its sex); Panel **b**: relationship between age (determined using skeletochronology) and right forelimb 2D:4D (variant II) for each sex; Panel **c**: relationship between age and left hindlimb 2D:4D for each sex; Panel **d**: relationship between age and left hindlimb 3D:4D for each sex. Age results were obtained from the second dataset containing only individuals from site 2 (*N* = 99; individuals after 3, 4, 5, 6, 7, or 8 hibernations. For more details, see Fig. S1)

No variation in digit ratio between three study sites was detected; accordingly, in the final mixed models, we did not take site of capture into account as a random variable (random intercept). No relationship was found between the size (SVL) of individuals and any digit ratio, except for right forelimb 2D:4D in the second dataset, i.e., the one in which the ages of toads were evaluated (Fig. 2, panel: a; Table S2). All obtained ICC values were significant and acceptable (ICC = 0.938, Table S1).

### Hindlimbs

Based on the GLM, we found that in the investigated toads, sex differences in digit ratio were significant only for left 2D:3D, with lower digit ratios in females (*F* = 8.638, *p* = 0.004), and for left 3D:4D, with lower digit ratios in males (*F* = 18.705, *p* < 0.001; Tables 2, S2). The effect sizes *g* ranged from 0.006 to 0.440 (Table 2). In the second dataset, the GLM showed that age was a significant variable for left hindlimb 2D:4D (*F* = 9.208, *p* = 0.003, Fig. 2, panel: c; Table S2) and 3D:4D (*F* = 6.664, *p* = 0.011; Fig. 2, panel: d; Table S2; for more demographic data, see Fig. S1), with lower values in older individuals (Fig. 2, panel: c, d). The t-test showed directional asymmetry (between left and right digit ratios) only in the case of 2D:3D, but the value of the effect size was 0.174, signifying that this effect was negligible, similar to those for other digit ratios on the hindlimbs (Table 3).

**Table 2:** Comparison of digit ratios (2D:3D, 2D:4D, and 3D:4D) on the forelimbs and hindlimbs between adult males and females of the common toad *Bufo bufo,* according to both forelimb digit numbering protocols (variants I and II). The last two columns present estimated absolute values of the effect size according to Hedges’ g statistics with magnitude categories

Variant	Digit ratio	Limb	Side		Males		Females	T–test			Hedges’ g	
				N	Mean ± SD	N	Mean ± SD	t	df	p	value	category
I	2D:3D^a^	Fore	L	**157**	**0.673 ± 0.067**	**136**	0.708 ± 0.065 ***	−4.611	285.9	< 0.001	0.538	medium
			R	**155**	**0.674 ± 0.062**	**138**	0.717 ± 0.078 ***	−5.289	262.55	< 0.001	0.625	medium
II	2D:3D	Fore	L	**157**	**1.271 ± 0.162**	**139**	1.184 ± 0.104 ***	5.537	269.83	< 0.001	0.627	medium
			R	157	1.187 ± 0.147	140	1.195 ± 0.161	−0.421	283.38	0.674	0.049	negligible
I	2D:4D	Fore	L	157	1.024 ± 0.154	139	1.035 ± 0.126	−0.728	292.12	0.467	0.084	negligible
			R	156	1.084 ± 0.196	140	1.081 ± 0.164	0.167	292.44	0.867	0.019	negligible
II	2D:4D	Fore	L	157	0.850 ± 0.089	136	0.836 ± 0.089	1.266	290.91	0.207	0.146	negligible
			R	**155**	**0.797 ± 0.106**	**137**	0.849 ± 0.089 ***	−4.585	289.45	< 0.001	0.531	medium
I	3D:4D	Fore	L	**157**	**1.526 ± 0.203**	**136**	1.461 ± 0.144 **	3.154	280.23	0.002	0.360	small
			R	**155**	**1.612 ± 0.258**	**137**	1.507 ± 0.171 ***	4.159	269.63	< 0.001	0.475	small
n/a	2D:3D	Hind	L	**157**	**0.709 ± 0.065**	**139**	0.690 ± 0.057 *	2.590	293.86	0.010	0.298	small
			R	157	0.695 ± 0.063	139	0.681 ± 0.077	1.635	268.44	0.103	0.192	negligible
n/a	2D:4D	Hind	L	157	0.413 ± 0.042	137	0.416 ± 0.035	−0.677	291.68	0.4989	0.078	negligible
			R	157	0.413 ± 0.048	138	0.414 ± 0.058	− 0.053	266.98	0.958	0.006	negligible
n/a	3D:4D	Hind	L	**158**	**0.584 ± 0.044**	**139**	0.605 ± 0.053 ***	−3.751	268.8	< 0.001	0.440	small
			R	158	0.591 ± 0.055	140	0.608 ± 0.060	−1.743	283.41	0.082	−0.203	small

^a^ 2D:3D in variant I corresponds to 3D:4D in variant II – for additional information, see Fig. 1; */**/*** indicates a significant difference between sexes, with levels of significance as follows: * – *p* < 0.05; ** – *p* < 0.01, *** – *p* < 0.001; n/a – not applicable

**Table 3:** Summary of t-tests for dependent variables comparing each digit ratio (2D:3D, 2D:4D, and 3D:4D) on the forelimbs and hindlimbs between the right and left body sides, according to both digit numbering protocols for the forelimbs (variants I and II), in the common toad *Bufo bufo.* The last two columns present estimated absolute values of the effect size according to Hedges’ g statistics with magnitude categories

Variant	Digit ratio	Limb	left	SD	right	SD	T–test			Hedges’ g	
							t	df	*P*	value	category
I	2D:3D^a^	Fore	0.689	0.068	0.695	0.071	1.078	290	*0.282*	0.081	negligible
II	2D:3D	Fore	1.230	0.144	1.189	0.150	3.515	295	***0.001***	0.281	small
I	2D:4D	Fore	1.030	0.141	1.084	0.180	4.320	294	***< 0.001***	0.335	small
II	2D:4D	Fore	0.844	0.098	0.821	0.102	2.922	290	***0.004***	0.225	small
I	3D:4D	Fore	1.497	0.181	1.563	0.228	−4.157	290	***< 0.001***	0.321	small
n/a	2D:3D	Hind	0.700	0.062	0.689	0.070	2.216	295	***0.027***	0.174	negligible
n/a	2D:4D	Hind	0.415	0.039	0.414	0.053	0.291	293	*0.771*	0.023	negligible
n/a	3D:4D	Hind	0.594	0.050	0.601	0.058	−1.830	296	*0.068*	0.141	negligible

^a^ 2D:3D in variant I corresponds to 3D:4D in variant II – for additional information, see Fig. 1; *n/a* Not applicable

### Forelimbs: variant I – reduced fifth digit

Based on the GLM, we found that the sex of the investigated toads was the only significant variable differentiating 2D:3D on both forelimbs, with lower digit ratios in males (left *F* = 22.393, *p* < 0.001; right *F* = 26.055, *p* < 0.001), and 3D:4D on both forelimbs, with lower digit ratios in females (left *F* = 10.082, *p* = 0.002; right *F* = 15.340, *p* < 0.001) (Tables 2, S2). In variant I, the effect sizes *g* ranged from 0.019 to 0.625 (Table 2). The GLM showed that, according to the second dataset, there was no relationship between the ages of individuals and any digit ratio (Table S2). The t-test showed directional asymmetry (between left and right digit ratios) in 2D:4D and 3D:4D, with effect sizes *g* ranging from 0.081 to 0.335 (Table 3).

### Forelimbs: variant II – reduced first digit

Based on the GLM, we found that the sex of the investigated toads was the only significant variable differentiating 2D:3D on the left forelimb, with lower digit ratios in females (*F* = 29.370, *p* < 0.001); 2D:4D on the right forelimb, with lower digit ratios in males (*F* = 19.088, *p* < 0.001); and 3D:4D on both forelimbs, with lower digit ratios in males (left *F* = 22.393, *p* < 0.001; right *F* = 26.055, *p* < 0.001) (Table 2, S2). It should be noted that 2D:3D in variant I corresponds to 3D:4D in variant II (see Fig. 1). In variant II, the effect sizes ranged from 0.049 to 0.627 (Table 2). In the second dataset, the GLM showed that age was a significant variable for right 2D:4D (*F* = 4.911, *p* = 0.029), as were sex (*F* = 17.494, *p* < 0.001) and, notably, body size (*F* = 4.817, *p* = 0.031; Fig. 2, panels: a, b; Table S2). In terms of age differences, the right forelimb 2D:4D of older individuals was lower (Fig. 2, panel: b). The t-test showed directional asymmetry (between left and right digit ratios) in 2D:3D and 2D:4D, with effect sizes ranging from 0.081 to 0.281 (Table 3).

**Fig. 3:**
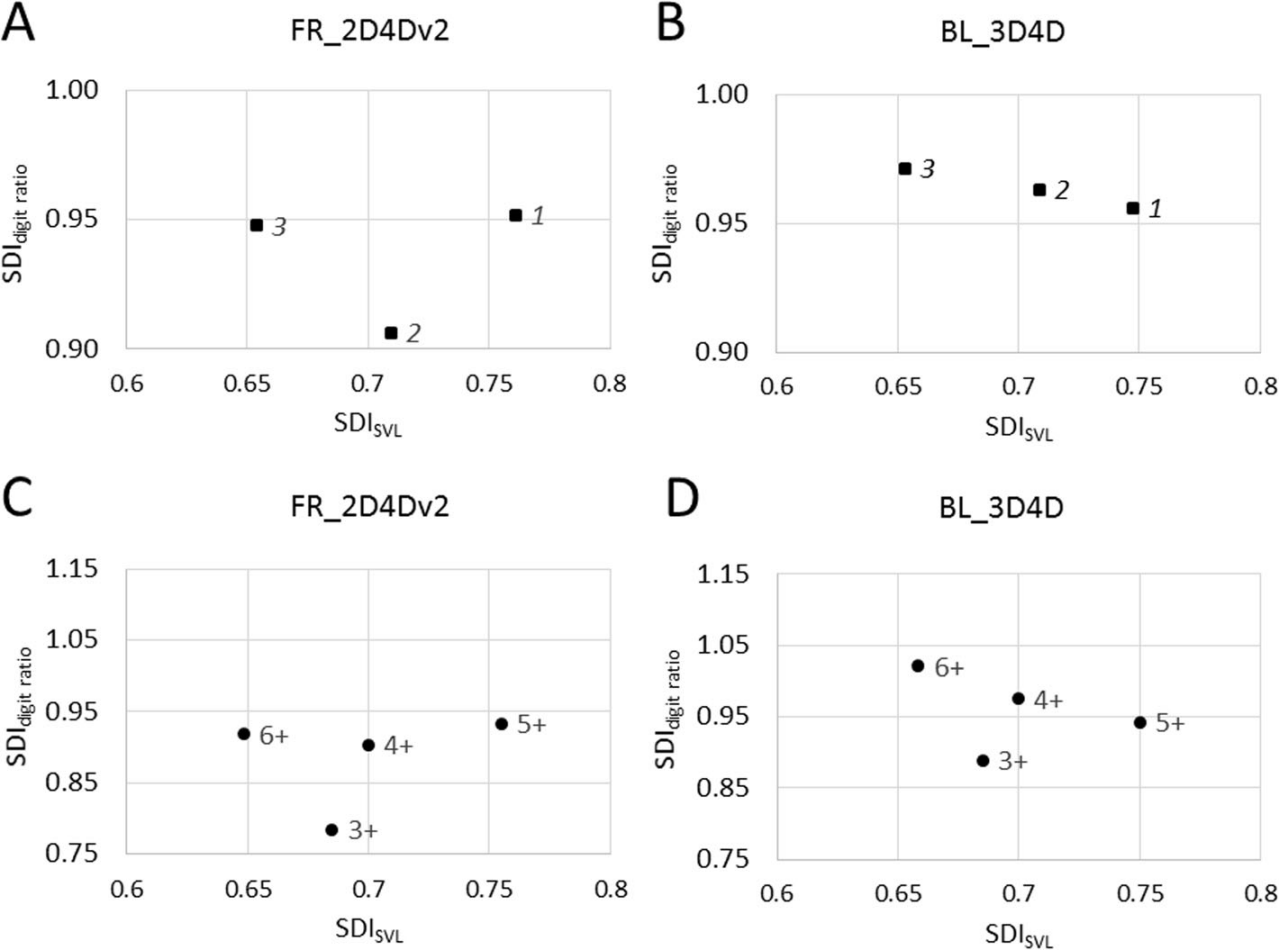
Phenotypic integration: relationship between the sexual dimorphism index (SDI) of body size (SDI_SVL_, snout-vent length) and digit ratio (SDI_digit ratio_) between three study sites marked as squares (panels: **a**, **b**) and four age cohorts marked as dots (panels: **c**, **d**). The digit ratio used was as follows: Panels **a**, **c** – right forelimb 2D:4D (variant II); Panels **b**, **d** – left hindlimb 3D:4D. Age results were obtained from the second dataset containing only individuals from site 2

The relationship between SDI_SVL_ and SDI_digit ratio_ for each site and age cohort is shown in Fig. 3 for right forelimb 2D:4D in variant II and left hindlimb 3D:4D (i.e., the digit ratios in which relationships with age and sex were detected in our models)*.* Due to the small number of surveyed populations and the existence of only 4 age classes (cohorts) in which the number of individuals exceeded 10 (Fig. S1), these data are presented only in chart form, without detailed statistical analysis.

## Discussion

In this study, we have described the current state of knowledge of digit ratios in amphibians and conducted a detailed analysis of the digit ratio patterns in the common toad *B. bufo*. The main question addressed here was whether any differences exist between males and females in the three most often investigated digit ratios, namely, 2D:3D, 2D:4D, and 3D:4D. The detected sex differences in digit ratio are quite ambiguous and do not form a consistent pattern. Significant differences between sexes were found in the following digit ratios: forelimbs, left 2D:3D (variants I and II), right 2D:3D (variant I), right 2D:4D (variant II), and left and right 3D:4D (variants I and II); hindlimbs, left 2D:3D and left 3D:4D (Tables 2, S2). However, the effect size values for these digit ratios corresponded to small or medium effects, similar to those detected in newts [17], whereas for comparison, [26] found medium or large effects in the studied anuran species. We detected no differences in hindlimb 2D:4D (Tables 2, S2). We have decided to present the full results for both variants of finger numbering, as we predict that, in future studies, other digit ratios will be used more frequently (e.g., 2D:5D, 3D:5D, and 4D:5D) [14]. We argue that variant II (i.e., the assumption of a reduced first digit, with digits II–V present on the forelimbs) should be commonly adopted in digit ratio studies in anurans (see: ***Limb and finger development in anurans***). It is impossible to provide a detailed discussion based on the results previously obtained by [25] in *O. pumilio* and by [5] in *Leptodactylus* frogs, as these authors analyzed only 2D:4D (excluding 2D:3D and 3D:4D); we detected no sex differences in this digit ratio when using their finger numbering scheme (referred to as variant I in this study). Nevertheless, we would like to draw attention to some general problems, which in our opinion are significant irrespective of the finger numbering system adopted. Clarification of the large discrepancies in the previously obtained results on amphibians is challenging (Table 1). First, the absence of sex differences in digit ratio may be due to the small sample sizes used in the research. Among the 12 species examined, only in the case of *E. pustulosus, L. podicipinus*, and *B. bufo* did the sample size exceed 100 individuals [5, 27] (the present study). Second, in the case of monomorphic species without clear sexual dimorphism, the lack of dimorphism in the digit ratio appears to be the expected state. This may be true of some frogs (*L. fuscus* and *L. pakeka*) and salamanders (*S. salamandra*) [5, 24, 28], as well as of members of other evolutionary lineages such as birds, e.g., the white stork *Ciconia ciconia* [44], or mammals, e.g., the American red squirrel *Tamiasciurus hudsonicus* [45]*.* However, it should be remembered that the lack of differences between sexes in the digit ratio may be a byproduct, or, in a case where individuals occupy different habitats, the result of natural selection (see Introduction, as well as [30]). Finally, digit ratio measurements may be susceptible to artificial variation resulting from the accuracy of the methods used or the condition of the preserved individuals [26, 46, 47]. [26] suggested that the differences in the results of the two studies on *O. pumilio* were related to differences in the measurement methodology adopted, i.e., hand calipers versus software analysis of digital photographs. Moreover, sample preservation may induce changes in digit ratio: in the case of New Zealand geckos *Woodworthia*, a change in the relative length of the phalanges was found in individuals following preservation for 1 year in 10% neutral buffered formalin [47]. This suggests that the optimal method entails the measurement of live individuals, whereas direct comparisons of digit ratios between preserved and live specimens should be avoided. Additionally, the current state of knowledge suggests that substantial interpopulational differences in digit ratio exist. This phenomenon was detected, e.g., in humans, and has been interpreted in the context of the impact of harsh environments [48]. As a consequence, interpopulational variability should be taken into account when designing research on digit ratio, whereas heretofore, in amphibians, only two studies have been conducted on more than one population: six populations in the case of *L. podicipinus* [5] and three in the case of *B. bufo* (this study). However, although our sampled populations came from two habitats that had been altered and one that was relatively uninfluenced by human activity, we found no interpopulational differences.

### Limb and finger development in anurans

We conducted our study using two alternative forelimb digit numbering schemes in Anura (Fig. 1), including one differing from those used in all of the earlier studies on the subject [24–27]. Our approach was based on a thorough review of the literature on the development of limbs in amphibians, which led us to call the previously implemented numbering of the digits of the forelimbs into question (referred to as variant I in this study). A general scheme of limb development in tetrapods, containing a description of the homologies of skeletal elements and based on a morphogenetic approach, was proposed by [49]. In general, postaxial limb development in anurans is analogous to that in amniotes [31]. However, the homology of amphibian digits and true digits of other tetrapods remains unclear [32, 50], since, in amphibians, digits develop through the differential proliferation of cells, whereas in amniotes such as mammals and birds, massive cell death of interdigital tissue is involved in the process [32, 50, 51]. [51] speculated that “ancestors of the modern amphibians and reptiles had cell death but the modern amphibian forms have lost it” or that the mechanism of cell necrosis during limb development was established after the amniotes had separated from the early amphibians. Additionally, limb development in Anura, which is associated with amphibian metamorphosis, occurs at a much later phylotypic stage than in amniotes [52]. Importantly, anurans, as mentioned in the Introduction, possess only four digits on their forelimbs; according to experimental studies, the lost finger is the most preaxial finger, i.e., finger I; as a consequence, anuran forelimbs contain fingers II–V [31–33]. The growth of fingers is a constant developmental sequence and takes place in the sequence IV, V, III, II [31]. In general, in all Anura, the fourth digit of the forelimb is the longest [50]. On the hindlimbs, the digits are formed in the sequence IV, III, V, II, I [31]. In addition to phalanges, some additional skeletal elements, such as prepollex (prepollices) and prehallux (prehallices, preaxial digit-like structures), may occur in anuran limbs, albeit with considerable interspecific variation [53]. We conclude that our approach, assuming that the first digit was reduced on anuran forelimbs (variant II), is strongly supported by earlier studies on limb development. Thus, the results concerning forelimbs in previous studies that adopted digit numeration according to [25] should be viewed with extreme caution [24–27].

### Directional asymmetry of the digit ratio and other issues

In our dataset, asymmetry was detected for most digit ratios on the forelimbs, except for 2D:3D in variant I (corresponding to 3D:4D in variant II) (Table 3). We found significant differences in digit ratios between body sides, but the calculated effect sizes were small regardless of the finger-numbering variant used, with *g* values clearly less than 0.5 (Table 3). In previous studies, asymmetry in digit ratio was also found on the forelimbs of *C. bransfordii* and *O. pumilio* and on the hindlimbs of *O. pumilio* [26]. Asymmetry in digit ratio is also present in other systematic groups: tailed amphibians [17], birds [9], and humans [54]. The direction of asymmetry differed between the variants of digit numbering employed. In variant I, the digit ratio showed right-biased asymmetry; in variant II, the opposite was true (Table 3). Another paper [1] suggested that right digit ratios are more closely correlated than left digit ratios with sex-dependent traits because the former are more susceptible to sex steroids. It is worth noting that in Anura, the process of forelimb emergence during metamorphosis varies between sides of the body. In our opinion, this may be linked with some differences in limb development, including digit ratio patterns. In anurans, one of the forelimbs growing during metamorphosis (Gosner stages 41 to 42) emerges through the spiraculum, and the second forelimb perforates the skin. In the case of *B. bufo*, it is the right forelimb that emerges first, regardless of the left-side position of the spiraculum [55]. Unfortunately, such detailed information on amphibian larval development is available for only a few species. Finally, the varying trajectories of forelimb development in anuran amphibians open up interesting possibilities for further research on digit ratios. Similarly, an interesting possibility is the inclusion of variation in locomotion modes in asymmetry research: toads and dendrobatids (e.g., *O. pumilio*) mainly move asymmetrically (walking, or, less frequently, jumping), whereas the other tested species are characterized by symmetrical locomotion (jumping and swimming).

Some studies indicate the phenotypic plasticity of amphibian limbs in various habitats. For example, *B. bufo* males from areas of intensive farmland were heavier and, importantly, were less symmetrical (in both hind- and forelimbs) than individuals from less disturbed sites, perhaps due to increased environmental stress during larval and/or postmetamorphic development [56]. To the best of our knowledge, variability in digit lengths and digit ratios has not been analyzed in relation to habitat quality. Our results, despite the large sample, do not fill this gap, as we found no differences between the study sites. The main limitation of our data in this context was our use of only three sampling sites (two sites under strong human pressure and one in a forested area, thus resembling the species’ original habitat much more closely). As a consequence, being aware of the limitations described above, we make no attempt to provide a more profound explanation of the detected directional asymmetry. In future research, it would be advisable to use other methods (e.g., fluctuating asymmetry) and to sample more sites covering a wider area.

The final issue is that lateralization exhibits a relationship with digit ratio [57]. Generally, hand preference in humans is a correlate of sensitivity to testosterone in the developing fetus [57], but [58] suggests that gene-based mechanisms mediate the effects of hand preference on digit ratios. In *B. bufo,* right-handedness was detected in 59% of individuals based on the snout-wiping test [59]. However, in the green toad *Bufotes viridis*, the opposite trend was detected, whereas in *R. marina*, as well as in true frogs, no dominance in forelimb use was observed (reviewed in [60]). Therefore, even for relatively closely related species such as bufonids, no compatibility is present in this trait. Moreover, [55] found no correlation between forelimb use preferences and the previously mentioned sequence of forelimb emergence in some anuran species (e.g., the common spadefoot toad *Pelobates fuscus* and the common frog *Rana temporaria*), whereas such a relationship has been detected in *B. bufo*. In our opinion, the observed asymmetry in anurans is due to asymmetrical development and/or, possibly, to variation in locomotion modes rather than being a derivative of lateralization. However, further research on the topic is required.

### Relationship between digit ratio and age

Using the second dataset (with individuals from site 2, the anthropogenically altered habitat), in which we determined individual age using skeletochronology, we found significant differences in right-forelimb 2D:4D; the relationship that we found was related to the age and size of the individual. In terms of age differences, older individuals appear to be characterized by a lower 2D:4D on the right forelimb (Fig. 2, panel: b). Interestingly, individuals at the age of 6 years exhibited both lower SDI_SVL_ values and higher SDI_digit ratio_ values than younger individuals (excluding those 5 years old; Fig. 3, panels: c, d). This can be interpreted as differences driven by environmental conditions during the tadpole stage or by selection during adulthood. Two scenarios may be considered in the interpretation of such results from wild populations. In the first scenario, individuals with advantageous hormonal milieus have a greater chance of survival in adulthood, which explains their overrepresentation in the older age cohorts. Notably, [61] showed that digit ratios are indicators of expected fitness and that early environmental effects coded in 2D:4D are of long-term relevance to reproductive success in the collared flycatcher *Ficedula albicollis*. In the second scenario, each age cohort exhibits specific traits resulting from the conditions in which they developed as tadpoles.

Generally, the digit ratio is sensitive to changes in the hormonal milieu (see experimental studies on the subject: [6, 8, 62]; thus, it may be used as a feature indicating environmental contamination with endocrine-disrupting substances. Amphibians are considered useful indicator organisms, especially for endocrine disruption [63, 64]. Therefore, their susceptibility to endocrine-disrupting substances during development appears certain; this applies to *B. bufo* as well [65–67]. As a consequence, digit ratio in anurans may be used as a bioindicator of some hormonal disorders, as suggested by [25]. This assumption was later confirmed experimentally with the use of testosterone added to water with developing tadpoles [5]. However, given the high level of sensitivity of amphibians to stress factors, it can be surmised that endocrine-disrupting substances are not the only factor capable of shaping the digit ratio pattern. An array of environmental factors, including variation in diet [68], competition [69, 70], the presence of predators [71, 72], diseases, and parasites, may also affect the growth trajectories and survival of metamorphs, potentially influencing adult fitness [73, 74]. Selection in juvenile amphibians is very strong, resulting in low overall survival rates at this stage; however, in temperate climates, conditions may vary from year to year, leading to different selective pressures between age cohorts. Depending on the stability of local habitat conditions, environmental factors may act with varying degrees of severity between seasons, e.g., in the form of water level fluctuations, drought, abnormally high temperatures, or varying pollution levels. As a consequence, each age cohort may show varied responses to stressors, depending on their severity and relevant interactions during development at the tadpole stage. These shifts in conditions may in turn be reflected in detectable differences in the digit ratio.

Thus, in our opinion, individuals of the same age should be used in future studies on digit ratios in amphibians in order to exclude differences between cohorts resulting from differences in developmental conditions at the time when limbs were formed. Accordingly, we recommend conducting further research under laboratory conditions, as obtaining a sufficient number of specimens of a particular age in natural conditions is expensive and time-consuming and necessitates injuring animals in the case of skeletochronology.

## Conclusion

Despite finding no clear pattern in digit ratio in our study, we highlight two issues that should be taken under consideration during further research: correct digit numbering on the forelimbs and ages of studied individuals.

After performing a careful study of the literature on the development of the finger on anuran forelimbs, we concluded that the correct sequence of finger numbering in Anura should be based on the assumption that it was the first digit that was reduced during phylogenesis; therefore, digits II–V are present on the forelimbs (as suggested by [31–33]). Thus, most of the published studies comparing digit ratios in Anura with those in Caudata and amniotes are invalid since the digits on the forelimbs are not homologous. Accordingly, the results of these studies should be interpreted with great caution. A deeper understanding of the mechanisms shaping the digit ratio in amphibians appears to be particularly important.

Despite using a relatively large sample, we were unable to find a clear male- or female-biased digit ratio pattern in the studied species using the approach described above (variant II - reduced first digit on the forelimbs). When considering significant differences for hindlimbs, we found the left 2D:3D digit ratio to be lower in females, whereas the left 3D:4D digit ratio was lower in males. In the case of forelimbs in variant II, the left 2D:3D digit ratio was significantly lower in females, while the right 2D:4D and the 3D:4D on both sides were significantly lower in males. This lack of a clear pattern indicates that the relationship between digit ratio and sex hormones is not straightforward in anurans, even in species with strong sexual dimorphism, such as the one used in our study. We detected no relationship between body size (SVL) and any of the studied digit ratios. However, in a subset of individuals where individual age had been determined (second dataset), the models indicated a relationship between age and the values of 2D:4D on the right forelimb.

The existence of variation in digit ratio correlated with individual age may represent a challenge for future research on the subject within anurans. Different conditions—and, as a consequence, different stress levels―during larval development (i.e., the tadpole stage) are capable of shaping a slightly different adult phenotype each year. Such shifts in environmental conditions may be reflected in detectable differences in digit ratios. Alternatively, variation in digit ratio between age cohorts may be a consequence of the improved survival of individuals with specific traits (possibly correlated with the digit ratio). In any case, further studies on the digit ratio in an applicational context, aimed at adapting and developing digit measurements as a simple, noninvasive feature for environmental monitoring, should take this variation into account, preferably using individuals of the same age from a given population.

## Supplementary Information

**Additional file 1: Figure S1.** Age structure of the common toad *Bufo bufo* based on the second dataset containing only individuals from site 2, with individuals after 3, 4, 5, 6, 7, or 8 hibernations (black columns – male; white columns – female). **Table S1.** Values of the intraclass correlation coefficient (ICC) calculated for 30 randomly selected individuals for which the digit measurements were carried out twice (D2 - D5, digit numbering according to variant II). **Table S2.** General linear model examining the effects of sex and size (SVL) (uneven models: M1, M3, …, M31) and the effects of age, prepared exclusively from the second dataset containing only individuals from site 2 (even models: M2, M4, …, M32), on digit ratios of the common toad *Bufo bufo*, according to both digit-numbering protocols for the forelimb (variants I and II, marked as “v2”).

## Data Availability

The datasets during and/or analyzed during the current study are available at https://data.mendeley.com/datasets/tytsxgmft4/1.
