# Supplementary material for: Digit ratio in the common toad Bufo bufo: the effects of reduced fingers and of age dependency
Source: Zoological Lett. 2021 Mar 25;7:5. doi: 10.1186/s40851-021-00174-y (PMC7992345; doi:10.1186/s40851-021-00174-y)
Supplement: Supplementary file 1 — Additional file 1: Figure S1. Age structure of the common toad Bufo bufo based on the second dataset containing only individuals from site 2, with individuals after 3, 4, 5, 6, 7, or 8 hibernations (black columns – male; white columns – female). Table S1. Values of the intraclass correlation coefficient (ICC) calculated for 30 randomly selected individuals for which the digit measurements were carried out twice (D2 - D5, digit numbering according to variant II). Table S2. General linear model examining the effects of sex and size (SVL) (uneven models: M1, M3, …, M31) and the effects of age, prepared exclusively from the second dataset containing only individuals from site 2 (even models: M2, M4, …, M32), on digit ratios of the common toad Bufo bufo, according to both digit-numbering protocols for the forelimb (variants I and II, marked as “v2”). [file 40851_2021_174_MOESM1_ESM.docx]

Supplementary materials

Figure S1. Age structure of common toad Bufo bufo based on second data set (“Site 2”), individuals after 3, 4, 5, 6, 7, 8 hibernations (black columns - male; white columns - female).

Table S1. Values of intraclass correlation coefficient (ICC) calculated for 30 randomly selected individuals that the digit measurements were carried out twice (D2 - D5, digits numbering according to Variant II).

| Digits | D2 | D3 | D4 | D5 |
| --- | --- | --- | --- | --- |
| *Forelimb* | 0.957 | 0.975 | 0.967 | 0.944 |
| *Hindlimb* | 0.932 | 0.953 | 0.839 | - |

Table S2. General linear model examining the effects of sex, size (SVL) (uneven models: M1, M3, …, M31) and examining the effects age - prepared only on second data set containing data from site 2 (even models: M2, M4, …, M32) on digit ratios of common toads *Bufo bufo*, according to both digits numbering protocols in forelimb (Variants I and II, marked as “**v2**”).

| Full data set | Estimate | S.E | F | P |  | Second data set | Estimate | S.E | F | P | |  |
| --- | --- | --- | --- | --- | --- | --- | --- | --- | --- | --- | --- | --- |
| *Front left limb* | | | | | | | | | | | | |
| M1: FL_2D3D |  |  |  |  |  | M2: FL_2D3D |  |  |  |  | |  |
| (Intercept) | 0.709 | 0.006 |  |  |  | (Intercept) | 0.689 | 0.041 |  |  | |  |
| SEX | -0.037 | 0.008 | 22.393 | **<0.001** |  | SEX | -0.046 | 0.016 | 7.773 | **0.006** | |  |
| SVL | <0.001 | 0.001 | 0.099 | 0.753 |  | SVL | -0.002 | 0.002 | 1.401 | 0.24 | |  |
|  |  |  |  |  |  | AGE | 0.005 | 0.008 | 0.312 | 0.578 | |  |
| M3: FL_2D3D**v2** |  |  |  |  |  | M4: FL_2D3D**v2** |  |  |  |  | |  |
| (Intercept) | 1.182 | 0.012 |  |  |  | (Intercept) | 1.137 | 0.085 |  |  | |  |
| SEX | 0.088 | 0.016 | 29.37 | **<0.001** |  | SEX | 0.076 | 0.034 | 5.046 | **0.027** | |  |
| SVL | 0.004 | 0.002 | 6.186 | 0.013 |  | SVL | 0.002 | 0.004 | 0.285 | 0.595 | |  |
|  |  |  |  |  |  | AGE | 0.014 | 0.017 | 0.718 | 0.399 | |  |
| M5: FL_2D4D |  |  |  |  |  | M6: FL_2D4D |  |  |  |  | |  |
| (Intercept) | 1.037 | 0.012 |  |  |  | (Intercept) | 0.89 | 0.076 |  |  | |  |
| SEX | -0.012 | 0.017 | 0.481 | 0.489 |  | SEX | 0.014 | 0.03 | 0.203 | 0.654 | |  |
| SVL | -0.002 | 0.002 | 0.886 | 0.348 |  | SVL | -0.002 | 0.003 | 0.286 | 0.594 | |  |
|  |  |  |  |  |  | AGE | 0.027 | 0.015 | 3.074 | 0.083 | |  |
| M7: FL_2D4D**v2** |  |  |  |  |  | M8: FL_2D4D**v2** |  |  |  |  | |  |
| (Intercept) | 0.836 | 0.009 |  |  |  | (Intercept) | 0.79 | 0.055 |  |  | |  |
| SEX | 0.013 | 0.012 | 1.304 | 0.254 |  | SEX | -0.011 | 0.022 | 0.258 | 0.613 | |  |
| SVL | 0.002 | 0.001 | 3.353 | 0.068 |  | SVL | -0.001 | 0.002 | 0.331 | 0.567 | |  |
|  |  |  |  |  |  | AGE | 0.014 | 0.011 | 1.583 | 0.212 | |  |
| M9: FL_3D4D |  |  |  |  |  | M10: FL_3D4D |  |  |  |  | |  |
| (Intercept) | 1.461 | 0.016 |  |  |  | (Intercept) | 1.299 | 0.098 |  |  | |  |
| SEX | 0.067 | 0.021 | 10.082 | **0.002** |  | SEX | 0.126 | 0.039 | 10.587 | **0.002** | |  |
| SVL | -0.002 | 0.002 | 0.869 | 0.352 |  | SVL | 0.002 | 0.004 | 0.311 | 0.579 | |  |
|  |  |  |  |  |  | AGE | 0.029 | 0.02 | 2.166 | 0.145 | |  |
| *Front right limb* | | | | | | | | | | |  |  |
| M11: FR_2D3D |  |  |  |  |  | M12: FR_2D3D |  |  |  |  | |  |
| (Intercept) | 0.717 | 0.006 |  |  |  | (Intercept) | 0.742 | 0.03 |  |  | |  |
| SEX | -0.043 | 0.008 | 26.055 | **<0.001** |  | SEX | -0.075 | 0.012 | 39.756 | **<0.001** | |  |
| SVL | <0.001 | 0.001 | 0.144 | 0.704 |  | SVL | 0.002 | 0.001 | 2.281 | 0.134 | |  |
|  |  |  |  |  |  | AGE | -0.004 | 0.006 | 0.361 | 0.549 | |  |
| M13: FR_2D3D**v2** |  |  |  |  |  | M14: FR_2D3D**v2** |  |  |  |  | |  |
| (Intercept) | 1.193 | 0.013 |  |  |  | (Intercept) | 1.308 | 0.068 |  |  | |  |
| SEX | -0.008 | 0.018 | 0.206 | 0.65 |  | SEX | 0.008 | 0.027 | 0.078 | 0.78 | |  |
| SVL | 0.003 | 0.002 | 2.796 | 0.096 |  | SVL | 0.004 | 0.003 | 1.505 | 0.223 | |  |
|  |  |  |  |  |  | AGE | -0.025 | 0.014 | 3.439 | 0.067 | |  |
| M15: FR_2D4D |  |  |  |  |  | M16: FR_2D4D |  |  |  |  | |  |
| (Intercept) | 1.081 | 0.016 |  |  |  | (Intercept) | 1.033 | 0.087 |  |  | |  |
| SEX | 0.004 | 0.021 | 0.033 | 0.855 |  | SEX | -0.061 | 0.034 | 3.1 | 0.082 | |  |
| SVL | 0.001 | 0.002 | 0.163 | 0.687 |  | SVL | 0.001 | 0.004 | 0.043 | 0.836 | |  |
|  |  |  |  |  |  | AGE | 0.018 | 0.017 | 1.051 | 0.308 | |  |
| M17: FR_2D4D**v2** |  |  |  |  |  | M18: FR_2D4D**v2** |  |  |  |  | |  |
| (Intercept) | 0.847 | 0.009 |  |  |  | (Intercept) | 0.965 | 0.05 |  |  | |  |
| SEX | -0.051 | 0.012 | 19.088 | **<0.001** |  | SEX | -0.084 | 0.02 | 17.494 | **<0.001** | |  |
| SVL | 0.002 | 0.001 | 3.712 | 0.055 |  | SVL | 0.005 | 0.002 | 4.817 | **0.031** | |  |
|  |  |  |  |  |  | AGE | -0.022 | 0.01 | 4.911 | **0.029** | |  |
| M19: FR_3D4D |  |  |  |  |  | M20: FR_3D4D |  |  |  |  | |  |
| (Intercept) | 1.509 | 0.019 |  |  |  | (Intercept) | 1.367 | 0.114 |  |  | |  |
| SEX | 0.103 | 0.026 | 15.34 | **<0.001** |  | SEX | 0.082 | 0.045 | 3.222 | 0.076 | |  |
| SVL | <0.001 | 0.003 | 0.032 | 0.858 |  | SVL | -0.004 | 0.005 | 0.549 | 0.46 | |  |
|  |  |  |  |  |  | AGE | 0.037 | 0.023 | 2.671 | 0.106 | |  |
| *Back left limb* | | | | | | | | | | | | |
| M21: BL_2D3D |  |  |  |  |  | M22: BL_2D3D |  |  |  |  | |  |
| (Intercept) | 0.689 | 0.005 |  |  |  | (Intercept) | 0.718 | 0.031 |  |  | |  |
| SEX | 0.021 | 0.007 | 8.638 | **0.004** |  | SEX | <0.001 | 0.012 | <0.001 | 0.995 | |  |
| SVL | 0.001 | 0.001 | 0.846 | 0.358 |  | SVL | 0.001 | 0.001 | 0.996 | 0.321 | |  |
|  |  |  |  |  |  | AGE | -0.005 | 0.006 | 0.549 | 0.461 | |  |
| M23: BL_2D4D |  |  |  |  |  | M24: BL_2D4D |  |  |  |  | |  |
| (Intercept) | 0.417 | 0.003 |  |  |  | (Intercept) | 0.479 | 0.02 |  |  | |  |
| SEX | -0.004 | 0.005 | 0.613 | 0.434 |  | SEX | -0.016 | 0.008 | 3.9 | 0.051 | |  |
| SVL | <0.001 | <0.001 | 0.488 | 0.485 |  | SVL | 0.001 | 0.001 | 0.688 | 0.409 | |  |
|  |  |  |  |  |  | AGE | -0.012 | 0.004 | 9.208 | **0.003** | |  |
| M25: BL_3D4D |  |  |  |  |  | M26: BL_3D4D |  |  |  |  | |  |
| (Intercept) | 0.608 | 0.004 |  |  |  | (Intercept) | 0.68 | 0.03 |  |  | |  |
| SEX | -0.024 | 0.006 | 18.705 | **<0.001** |  | SEX | -0.025 | 0.012 | 4.591 | **0.035** | |  |
| SVL | <0.001 | 0.001 | 0.096 | 0.757 |  | SVL | <0.001 | 0.001 | 0.008 | 0.927 | |  |
|  |  |  |  |  |  | AGE | -0.015 | 0.006 | 6.664 | **0.011** | |  |
| *Back right limb* | | | | | | | | | | | | |
| M27: BR_2D3D |  |  |  |  |  | M28: BR_2D3D |  |  |  |  | |  |
| (Intercept) | 0.681 | 0.006 |  |  |  | (Intercept) | 0.629 | 0.042 |  |  | |  |
| SEX | 0.014 | 0.008 | 2.819 | 0.094 |  | SEX | 0.014 | 0.017 | 0.685 | 0.41 | |  |
| SVL | <0.001 | 0.001 | 0.008 | 0.928 |  | SVL | 0.002 | 0.002 | 0.761 | 0.385 | |  |
|  |  |  |  |  |  | AGE | 0.01 | 0.008 | 1.267 | 0.263 | |  |
| M29: BR_2D4D |  |  |  |  |  | M30: BR_2D4D |  |  |  |  | |  |
| (Intercept) | 0.414 | 0.005 |  |  |  | (Intercept) | 0.411 | 0.03 |  |  | |  |
| SEX | <0.001 | 0.006 | 0.004 | 0.948 |  | SEX | -0.005 | 0.012 | 0.145 | 0.704 | |  |
| SVL | -0.001 | 0.001 | 1.962 | 0.162 |  | SVL | 0.001 | 0.001 | 0.194 | 0.661 | |  |
|  |  |  |  |  |  | AGE | -0.002 | 0.006 | 0.073 | 0.788 | |  |
| M31: BR_3D4D |  |  |  |  |  | M32: BR_3D4D |  |  |  |  | |  |
| (Intercept) | 0.609 | 0.005 |  |  |  | (Intercept) | 0.647 | 0.031 |  |  | |  |
| SEX | -0.011 | 0.007 | 2.875 | 0.091 |  | SEX | -0.018 | 0.012 | 2.189 | 0.142 | |  |
| SVL | -0.001 | 0.001 | 2.063 | 0.152 |  | SVL | <0.001 | 0.001 | 0.057 | 0.812 | |  |
|  |  |  |  |  |  | AGE | -0.01 | 0.006 | 2.678 | 0.105 | |  |
